# Success

**Published:** 1874-12

**Authors:** 


### Editorial.

### SUCCESS.

In the every day affairs of life various meanings are given to the word success.

It is often said, of one who has made money, he has been successful; and this is generally expressed regardless of the means or methods by which it is attained. But in the true and proper sense, he only has been successful who has made money in an honorable and useful way.

Of the dentist it is sometimes said, he has been successful, simply because he has made money, though he has prostituted his profession and used it merely for that purpose doing everything simply with the object of making the most money.

Some in our profession accomplish just this and nothing more. Fortunately those who start out in the dental profession with this intent are comparatively few. But there are some who practice solely for the purpose of gain, and the consequence is that almost everything they do is either productive of no good to their patients, or results in positive injury.

The chief and highest aim of the physician, either in a general or special way, should be to conserve the health and physical well being of those committed to his care, and to relieve and remove these sufferings and diseases to which so many are subject. Pecuniary reward is, essentially, and ought always to be regarded as a secondary consideration and he who trifles with the health and life of those committed to his charge is guilty of a crime that somewhere will receive its equivalent.

Can such a one though he makes money, be truly said to be successful?

Success should only be attributed to him who accomplishes the greatest good of which he is capable, from pure and exalted motives and desires. Would the surgeon who either kills or permits to die the large majority of his patients, though he might receive large fees, be regarded as successful?

Would the physician who should loose three-fourths of his patients, though he might accumulate a fortune, be esteemed successful?

Should the dentist who sacrifices thousands of natural teeth that could be made comfortable and serviceable for perhaps a lifetime, or treats teeth that are diseased in a careless slipshod manner that will not serve an iota for their preservation or welfáre, be regarded as successful even though he should save money?

On the other hand should he who contributes, to the best of his ability, to the preservation of health, comfort and usefulness of the teeth committed to his care, and secures the highest results possible in every case be regarded as a failure, even though he does not accumulate money? He is in the truest and highest sense the successful man.

